# Large mediastinal germ cell tumor with disease progression during systemic therapy

**DOI:** 10.1093/jscr/rjab416

**Published:** 2021-09-30

**Authors:** Diana S Hsu, Sawley A Wilde, Kian C Banks, Jeffrey B Velotta

**Affiliations:** Department of Surgery, Highland Hospital, University of California, San Francisco - East Bay, Oakland, CA 94602, USA; Thoracic Surgery, Kaiser Oakland Medical Center, Oakland, CA 94611, USA; Department of Surgery, Highland Hospital, University of California, San Francisco - East Bay, Oakland, CA 94602, USA; Thoracic Surgery, Kaiser Oakland Medical Center, Oakland, CA 94611, USA; Department of Surgery, Highland Hospital, University of California, San Francisco - East Bay, Oakland, CA 94602, USA; Thoracic Surgery, Kaiser Oakland Medical Center, Oakland, CA 94611, USA; Thoracic Surgery, Kaiser Oakland Medical Center, Oakland, CA 94611, USA

**Keywords:** mediastinal germ cell tumor, immature teratoma, neoadjuvant therapy, multidisciplinary management, complex resection

## Abstract

A 29-year-old male developed acute onset severe shortness of breath and fevers and was found to have a 17 cm anterior mediastinal mass with immature teratoma and possible mixed germ cell tumor on biopsy. He remained hospitalized during neoadjuvant cisplatin-based chemotherapy due to compressive symptoms from his mass and neutropenic fevers. Despite 3 cycles of therapy, his tumor mildly increased in size. After multidisciplinary discussion, he underwent urgent en bloc resection with a right hemi-clamshell incision. His postoperative course was uncomplicated and he was discharged to home within a week. His final pathology demonstrated mixed germ cell tumor.

## INTRODUCTION

Mediastinal germ cell tumors (GCT) are uncommon, and of these, immature teratomas are even more rare, comprising 1.8% of all mediastinal teratomas. These tumors are more prevalent in males and typically are diagnosed before the age of 40 [1]. Survival is worse in patients with mediastinal versus testicular GCT and for those with metastatic disease and beta HCG elevation at the time of diagnosis. Five-year survival rate for patients with non-seminomatous mediastinal GCT is 30-45% [2]. GCTs are usually chemosensitive and first-line therapy is cisplatin-based chemotherapy, followed by surgery [3].

We report herein a case of chemotherapy-resistant immature teratoma in a patient who was unable to be discharged from the hospital due to compressive symptoms of his large mediastinal mass.

## CASE REPORT

**Figure 1:**
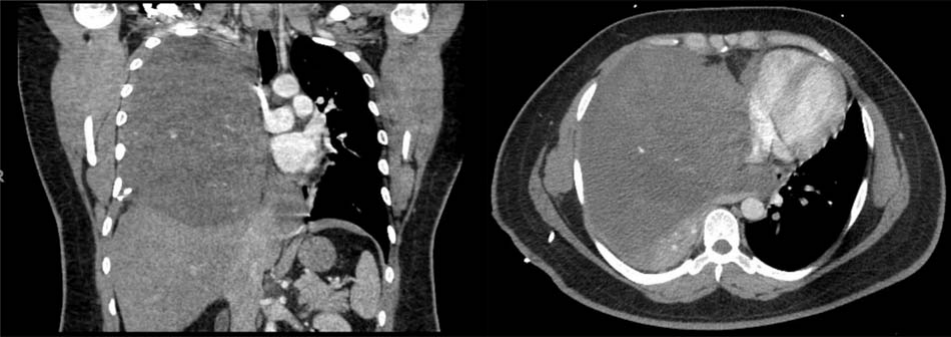
(Left to right) Coronal and axial images of 17 × 16 × 17.3 cm heterogeneous anterior mediastinal mass with compression of the right lung and left-sided mediastinal shift.

A 29-year-old male with history of Kawasaki’s disease presented with 1 week of cough, dyspnea on exertion, weight loss and fevers. His workup revealed a WBC of 13 K/μl (ref. 3.7–11.1 K/μl) and a 17 × 16 × 17.3 cm heterogenous mass with calcifications in the right hemithorax causing compression of the right lung and bronchus and left-sided mediastinal shift (Fig. 1). An echocardiogram demonstrated compression of the right heart from his mediastinal mass with a normal ejection fraction.

Pleural effusion analysis was negative for malignancy and cultures were negative. Ultrasound-guided biopsy of his mediastinal mass demonstrated immature teratoma with focal nonspecific AFP stain, and thus mixed GCT was unable to be ruled out. Scrotal ultrasound and metastatic workup were negative for other lesions. His admission serum markers were significant for AFP of 5756 ng/ml (ref 0.0–8.3 ng/ml) and LDH 343 U/l (ref <270 U/l). His beta HCG was within normal limits. He remained as an inpatient for his entire workup and eventual chemotherapy due to significant oxygen requirements and inability to mobilize or lay supine.

He was started on a chemotherapy regimen of cisplatin, etoposide and ifosfamide (VIP) as an inpatient. His follow-up imaging demonstrated increase in mass size to 18 × 16 × 17 cm with increasing mass effect despite improvement of his serum tumor markers—AFP 243 ng/ml, LDH 347 U/l—at the end of 3 cycles. He continued to require inpatient telemetry admission throughout his neoadjuvant therapy due to neutropenic fevers, pancytopenia and compressive symptoms from his mass.

After multidisciplinary tumor board discussion and consultants within national cancer centers, there were differing opinions regarding further chemotherapy versus surgical resection.

**Figure 2:**
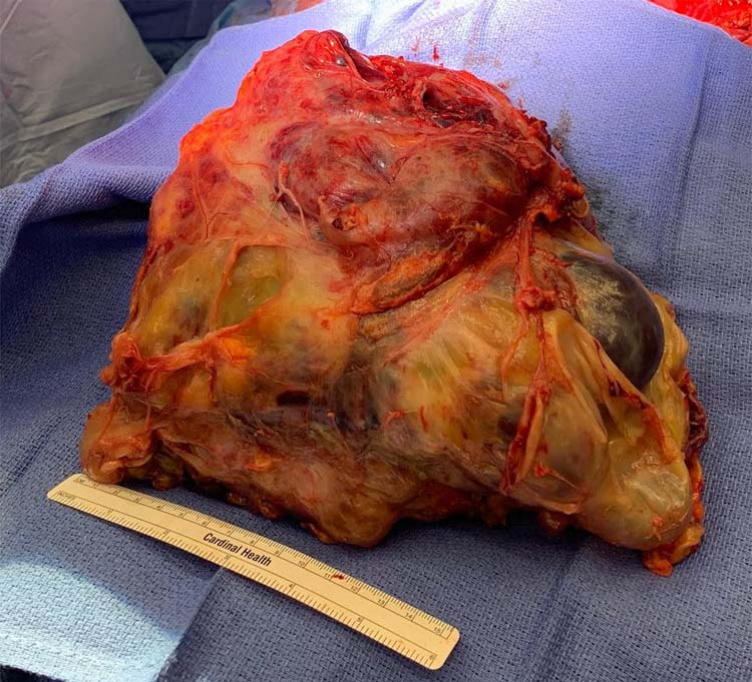
Tumor specimen. Final pathology demonstrated a mixed GCT with mostly teratoma. Weight: 4.6 kg.

Our facility, which is a major regional cancer center, was consulted and recommended surgical resection, since his symptoms persisted and the tumor increased in size despite chemotherapy. He was transferred to our facility and underwent urgent resection with cardiopulmonary bypass on standby. His operation consisted of median sternotomy and right hemi-clamshell incision for mass and right middle lobe en bloc resection. The mass was compressing, but not invading, the heart or pericardium. No other lesions were identified. The patient received 3 units of packed red blood cells and 3 units of fresh frozen plasma intraoperatively. Estimated blood loss was almost 2 l. Final specimen weight was 4.6 kg. His final pathology was mixed GCT with a predominant teratoma component. There was a positive margin at the staple line of the right middle lobe wedge resection (Fig. 2).

The patient was extubated in the operating room and transferred to the intensive care unit for close monitoring. He was transferred out of the unit on postoperative day 1. The remaining postoperative course was uncomplicated and he was discharged on postoperative day 6 with no oxygen requirements and good mobility.

## DISCUSSION

This patient ultimately had a large mixed GCT not responsive to chemotherapy. Unlike mature mediastinal teratomas, immature teratomas behave aggressively, particularly in adult patients, and generally are not responsive to chemotherapy. Thus, complete surgical resection is crucial [4]. Hiroshima *et al*. concluded that complete resection after neoadjuvant therapy gave patients a favorable prognosis [5]. The pediatric literature on extracranial immature teratomas has concluded that these should be treated with surgery alone [6]. Arai *et al*. identified that patients, such as ours, who have increased tumor size despite decreasing tumor marker levels pose a challenge in determining whether systemic therapy is effective. This is possibly due to changing tumor characteristics and the rare somatic-type malignant transformation within the tumor during neoadjuvant therapy [3, 7]. Teratomas with malignant transformation have a worse prognosis with high recurrence rates and poor survival [8]. This patient did not have a malignant transformation on final pathology, but his final diagnosis of mixed GCT likely explains why his tumor marker levels decreased without a response in tumor size over the course of neoadjuvant therapy. In addition, this patient’s clinical course is similar to the documented phenomenon of ‘growing teratoma syndrome’ where non-seminomatous GCTs continue to increase in size while receiving systemic therapy and eventually patients require urgent surgery before completion of neoadjuvant chemotherapy [9].

This patient’s tumor was much larger than the reported average mediastinal GCT of 10 cm and 415 g [10]. We started with a median sternotomy to fully evaluate the extent of association with the pericardium and superior vena cava and then extended into a right hemi-clamshell thoracotomy based on intraoperative findings. This exposure proved to be effective and adequate and has been used with success at other institutions [11]. We also recommend when performing this complex surgery with high morbidity and mortality, that cardiopulmonary bypass be on immediate standby in the operating room, which we did in this case. Due to the overall complex nature of this particular cancer and multiple specialties needing to be involved, we recommend that these surgeries also be performed at high volume, specialized cancer centers.

Mediastinal GCTs in adults are extremely rare. When these malignancies do not respond appropriately to systemic therapy, we recommend immediate radical surgical excision be performed at regional centers of excellence with adequate perioperative support.

## CONFLICT OF INTEREST STATEMENT

No conflicts of interest.
